# FROM GAINS TO DECLINE: EFFECTS OF STRUCTURED PRE-DIALYTIC EXERCISE TRAINING AND DETRAINING ON PHYSICAL FITNESS, QUALITY OF LIFE, AND INFLAMMATION IN HAEMODIALYSIS PATIENTS

**DOI:** 10.2340/jrm.v57.44067

**Published:** 2025-10-22

**Authors:** Shu-Chun HUANG, Ji-Tseng FANG, Yu-Chieh HUANG, Chun-Yueh LIN, Ching-Chung HSIAO

**Affiliations:** 1 Department of Physical Medicine and Rehabilitation, New Taipei Municipal Tucheng Hospital, Chang Gung Memorial Hospital, Taipei; 2 Department of Physical Medicine & Rehabilitation, Chang Gung Memorial Hospital, Linkou, Taoyuan; 3 College of Medicine, Chang Gung University, Taoyuan; 4 Department of Nephrology, Chang Gung Memorial Hospital, Linkou, Taoyuan; 5 Department of Physical Therapy, College of Medical and Health Science, Asia University, Taichung; 6 Department of Medical Education, Chang Gung Memorial Hospital, Linkou, Taoyuan; 7 Department of Nephrology, New Taipei Municipal Tucheng Hospital, Chang Gung Memorial Hospital, New Taipei City, Taiwan

**Keywords:** body composition, cardiorespiratory fitness, haemodialysis, muscle strength, peak oxygen consumption

## Abstract

**Objective:**

Pre-dialysis exercise training may benefit haemodialysis patients, but the effects of structured aerobic and resistance training remain unclear. This study evaluated the effects of a 6-month training performed 1–2 h before haemodialysis on muscle strength, cardiorespiratory fitness, quality of life, and systemic inflammation.

**Design:**

A 3-phase self-controlled design: 3-month control, 6-month aerobic and resistance training (72 sessions) and nutrition counselling, and 3-month follow-up.

**Patients:**

Haemodialysis patients > 3 months.

**Methods:**

Assessments were performed every 3 months at 5 time points. Outcomes included physical fitness (dual-energy X-ray absorptiometry, isokinetic dynamometry, and cardiopulmonary exercise testing), Kidney Disease QOL questionnaire, International Physical Activity Questionnaire, nutrition, and plasma pro- and anti-inflammatory cytokines.

**Results:**

Of 118 patients screened, 29 entered training and 22 completed 72 sessions (92% compliance). Pre-dialysis exercise training improved muscle mass, cardiorespiratory fitness, quadriceps strength, physical and mental components, and disease-specific quality of life; cardiorespiratory fitness and strength declined at 3-month follow-up, but muscle mass remained. Cytokine levels were unchanged, suggesting minimal pro- or anti-inflammatory effects.

**Conclusion:**

A 6-month pre-dialysis exercise programme improved cardiorespiratory fitness, strength, muscle mass, and quality of life with high compliance. It may be viable for haemodialysis patients, though maintenance is needed to sustain benefits.

**ClinicalTrials.gov ID:**

NCT05649657

## LAY ABSTRACT

People receiving maintenance haemodialysis often have low endurance, weak muscles, and reduced quality of daily life, leading to poorer health outcomes. While exercise during or on non-dialysis days has shown benefits, little is known about exercising just before dialysis. In this study, patients joined a 6-month supervised programme combining aerobic and strength exercises before dialysis. The programme improved endurance, strength, muscle mass, and quality of life. Most gains faded within 3 months after training ended, except muscle mass, which was maintained. Exercise before dialysis did not change blood markers of inflammation, suggesting minimal pro- or anti-inflammatory effects. Overall, this approach was effective, and easy to follow, providing a practical alternative to existing exercise models. Sustained strategies are needed to preserve the benefits.

Patients receiving maintenance haemodialysis (HD) account for over 0.04% of the global population and contribute to an increasing burden on healthcare systems (1). Among individuals under maintenance HD, physical fitness deterioration and chronic fatigue are well-documented conditions (2). Notably, low cardiorespiratory fitness (CRF) and sarcopenia are significant predictors of cardiovascular events and mortality (3, 4), and are also associated with reduced health-related quality of life (QOL) (5). Patients on maintenance HD are generally sedentary (6); exercise therapy has been suggested to improve cardiovascular morbidity and mortality in this population (7).

Regarding the timing of exercise training, 2 approaches are commonly used: exercise during haemodialysis (HD) and on non-HD days. The protocols of the former primarily consist of supine or seated exercises, such as aerobic cycling or resistance exercises using Thera-bands or weighted ankle cuffs (8). Exercise training on non-dialysis days offers advantages, including unrestricted upper limb movement, higher-intensity training, and a lower risk of catheter dislodgement, bleeding, and hypotension. However, hospital-based protocols on non-HD days exhibited a higher dropout rate (9).

Only a few studies have investigated hospital-based exercise training prior to haemodialysis (HD). To the best of our knowledge, 5 relevant studies have been reported. Four focused exclusively on resistance training (RT) (10–13), and 1 evaluated Wii Fit Plus, a virtual reality programme encompassing various exercise modalities, such as hula-hoop, dance, juggling, and one-arm pull back (14). However, none of the studies investigated a conventional structured aerobic and resistance training, nor did they include follow-up evaluations after training discontinuation. Additionally, only 1 study assessed the effects of training on systemic inflammation (13), and 2 studies assessed QOL (10, 12). Please refer to the Discussion for details.

Haemodialysis patients often exhibit chronic inflammation, impairing muscle protein synthesis and affecting body composition and function (15). While exercise may reduce inflammation (16), the effect of pre-dialytic exercise – performed in a hypervolemic state – on systemic inflammation, either positively or negatively, remains unclear.

This study aimed to evaluate the effects of pre-dialytic exercise (PDE) conducted 1–2 h before HD on muscular and cardiorespiratory fitness, QOL, and inflammatory cytokines. A within-subject design included a 3-month control, 6-month training, and 3-month follow-up. We hypothesized that PDE would improve CRF, quadriceps strength, body composition, QOL, and systemic inflammation. Gold-standard measurements of physical fitness including body composition analysis using dual-energy X-ray absorptiometry (DXA), isokinetic dynamometry (IsoK) and cardiopulmonary exercise test (CPET) were employed. Additionally, a 3-month post-training follow-up assessed carry-over effects.

## MATERIALS AND METHODS

### Study protocol

Participants were eligible for inclusion if they had been undergoing HD for more than 3 months, were over 20 years of age, had adequate dialysis (Kt/V > 1.2), obtained approval from their nephrologist, and were able to walk independently for more than 10 m. Exclusion criteria included recent hyperkalaemia, medical or orthopaedic conditions, psychological disorders (17), and a history of heart failure or inability to participate in cycling or exercise testing. This study was approved by the Chang Gung Medical Foundation Institutional Review Board. It was conducted between July 2020 and February 2025. All participants provided written informed consent after the researchers explained the experimental procedures. The study complied with the tenets of the Declaration of Helsinki.

**Fig. 1:**
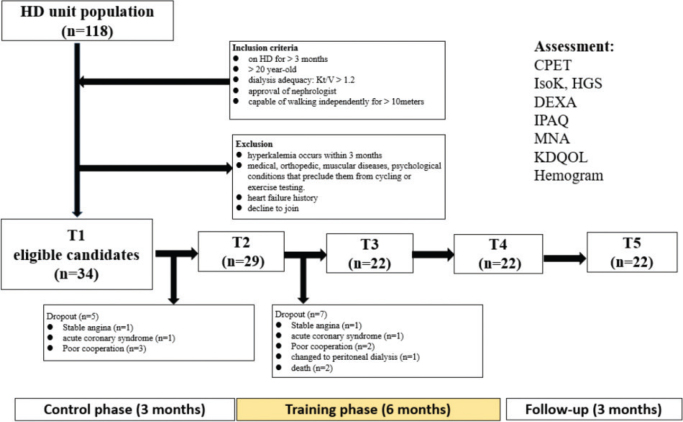
**Flowchart depicting the full course of patient recruitment, exclusion criteria application, and dropout during the trial**. Each participant was assessed at 5 time points (T1–T5) at ~3-month intervals. The control phase (T1–T2) lasted 3 months with usual care only. The training phase (T2–T4) spanned 6 months and included exercise training and nutrition education. The follow-up phase (T4–T5) covered 3 months, monitoring changes after training cessation under standard care. CPET: cardiopulmonary exercise testing; DXA: dual-energy X-ray absorptiometry; HGS: hand grip strength; IsoK: isokinetic dynamometry; IPAQ: International Physical Activity Questionnaire; KDQOL: Kidney Disease Quality of Life; MNA: mini-nutritional assessment.

This study employed self-controlled design. Over the study period, each participant underwent assessments at 5 time points (T1, T2, T3, T4, and T5) with about 3 months between each time point. Between T1 and T2 was the control phase, when the participants received usual medical care only. Between T2 and T4 was the training phase, during which exercise training and nutrition education were implemented. The T4–T5 follow-up phase monitored changes after training cessation with standard medical care only (Fig. 1).

The assessment involved CPET, IsoK, hand grip strength (HGS), DXA, mini-nutritional assessment (MNA), generic and CKD-specific QOL (Kidney Disease Quality of Life, KDQOL), International Physical Activity Questionnaire (IPAQ), plasma inflammatory cytokine and white blood cell differential counts (see Fig. 1).

### Exercise training programme

Before training, an exercise physiologist assessed safety risk profiles and provided an exercise prescription. This was then carried out by a properly trained physical therapist. Contraindications for exercise training primarily followed the guidelines of the American College of Sports Medicine. Common conditions included a resting sinus heart rate > 100 bpm and blood pressure > 180/110 mmHg (18). Blood pressure and ECG were monitored throughout each session. The participants visited the rehabilitation centre twice or three times weekly for about 72 sessions in total, which lasted between 24 and 36 weeks. The exercise prescription comprised cyclic aerobic and resistance training. The training was performed 1–2 h before HD. Cycle ergometry (Lode Corival V3; Lode BV, Groningen, the Netherlands), recumbent stepper (NuStep; NuStep LLC, Plymouth, MI, USA), and treadmill (Biodex; Biodex Medical Systems, Shirley, NY, USA) were used. The intensity was set initially at ventilatory anaerobic threshold (VAT) based on CPET. The duration was 30 min per session, plus 3-min warm-up and 3-min cool-down. Once the patient was able to tolerate intensity at VAT for 20 min continuously, high-intensity interval training (HIIT) was implemented. The training intensity was given initially at 40% peak work rate (low) for 2 min and followed by 80% peak work rate (high) for another 2-min interval. The intensity changed repeatedly at high and low intensity throughout the session. Brief pauses were allowed during a single training session. If the patient could complete a single session without any pause, the high intensity of HIIT was adjusted to increase by up to 5% (for example, 80% to 84%) (19). Additionally, cyclic RT was performed using an isokinetic training system (BodyGreen; B.Green Technology Co, Ltd, Xiushui Township, Raiwan, ROC). Eight devices were applied: leg press, thigh adduction/abduction, leg extension/curl, shoulder press/pull down, pec dec/fly, wait twist, chest press or seated rowing. In each session, 3 devices were used – typically 1 targeting the upper limbs, 1 the lower limbs, and 1 the trunk. Ten repetitions were performed on each device, with participants verbally instructed to exert maximal volitional effort. Rest intervals between repetitions and between exercises were self-selected by participants to avoid undue fatigue. In principle, resistance training was performed prior to aerobic training.

Please refer to Appendix S1 for detailed information on methodology: 2.3. Nutrition program; 2.4. Cardiopulmonary exercise testing; 2.5. Body composition; 2.6. Isokinetic dynamometry; 2.7. Hand grip strength; 2.8. Hong Kong Chinese Kidney Disease Quality of Life; 2.9 International Physical Activity Questionnaire (IPAQ); 2.10. Measurement of plasma inflammatory cytokines and white blood cell differential counts; 2.11. Nutritional assessment.

### Statistics

The values were expressed as median (1st quartile, 3rd quartile). Various parameters across 5 time points (T1 to T5) were analysed using a mixed model of repeated measurements. Normality of residuals for variables with significant findings in the mixed-model repeated measures analysis was tested using the Kolmogorov–Smirnov test, supplemented by Q–Q plots and histograms with normal curves for representative variables. The analysis used stage as a fixed factor and subject ID as a random factor, with a variance components covariance structure. Parameters were estimated by restricted maximum likelihood, and Bonferroni correction was applied for multiple comparisons of the main effects of stage. The overall type 3 fixed effect of staging (T1 to T5) and pairwise comparison were also calculated. The significance was set at *p*-value less than 0.05. Data were analysed using SPSS version 28.0 (IBM Corp, Armonk, NY, USA).

## RESULTS

A total of 118 individuals from the HD units at New Taipei Municipal TuCheng Hospital and Linkou Chang Gung Memorial Hospital, Taiwan were screened. After applying inclusion and exclusion criteria, 34 participants were enrolled and completed the initial T1 assessment. During the control phase, 5 dropped out, including 2 due to cardiac events. During the training phase (T2–T3), 7 additional participants withdrew, including 2 due to cardiac events and 2 deaths, one from respiratory failure secondary to hypertensive crisis-related acute pulmonary oedema and the other from septic shock.

Twenty-nine participants entered T2, and 22 completed the 72-session training at T4. After excluding 5 dropouts due to medical conditions, the compliance rate for the 6-month training was 92% (22/24) (see Fig. 1). Among those who completed T6, the median age was 62.0 years, BMI 24.4, and the most common comorbidities were hypertension and diabetes. The median Kt/V was 1.730 (Table I).

**Table I:** Basic information

Sex (F/M), *n*	7/15
Age, years, median (IQR)	62.0 (51.5, 69.25)
Height, cm, median (IQR)	163 (157, 170)
BMI, kg/m^2^, median (IQR)	24.4 (22.0, 27.8)
Duration of haemodialysis, months, median (IQR)	27 (7, 74)
Comorbidity, *n* (%)	
Hypertension	13 (59%)
Diabetes	10 (45%)
Hyperlipidaemia	7 (32%)
Hyperuricemia	4 (18%)
Arrhythmia	2 (9%)
Stroke	2 (9%)
PAOD	1 (5%)
Asthma	1 (5%)
Malignancy	1 (5%)
Liver disease	1 (5%)
Cardiothoracic ratio, median (IQR)	0.485 (0.440, 0.540)
Kt/V, median (IQR)	1.730 (1.355, 2.592)
Urea reduction ratio, median (IQR)	0.74 (0.70, 0.78)
Haemoglobin, g/dL, median (IQR)	10.5 (10.0, 11.6)
Albumin, g/dL, median (IQR)	3.98 (3.72, 4.10)
Calcium, mg/dL, median (IQR)	9.1 (8.8, 9.6)
Phosphate, mg/dL, median (IQR)	5.3 (4.5, 6.5)
iPTH, pg/mL, median (IQR)	168 (95, 427)
Sodium, mEq/L, median (IQR)	136 (135, 137)
Potassium, mEq/L, median (IQR)	4.7 (4.3, 5.3)
Beta-blocker	10 (45%)
CCB	8 (36%)
ARB	5 (23%)
ACEi	2 (9%)
Diuretics	1 (5%)
Nitrates	1 (5%)
Antidiabetic medication, *n* (%)	
DPP-4 inhibitor	4 (18%)
Insulin	2 (9%)
Sulfonylureas	1 (5%)
Meglitinide	1 (5%)

ACEi: angiotensin converting enzyme inhibitor; ARB: angiotensin II receptor blocker; BMI: body mass index; CCB: calcium channel blocker; DPP-4 inhibitor: dipeptidyl peptidase-4 inhibitor; F: female; iPTH: intact parathyroid hormone; M: male; PAOD: peripheral arterial occlusion disease.

**Fig. 2:**
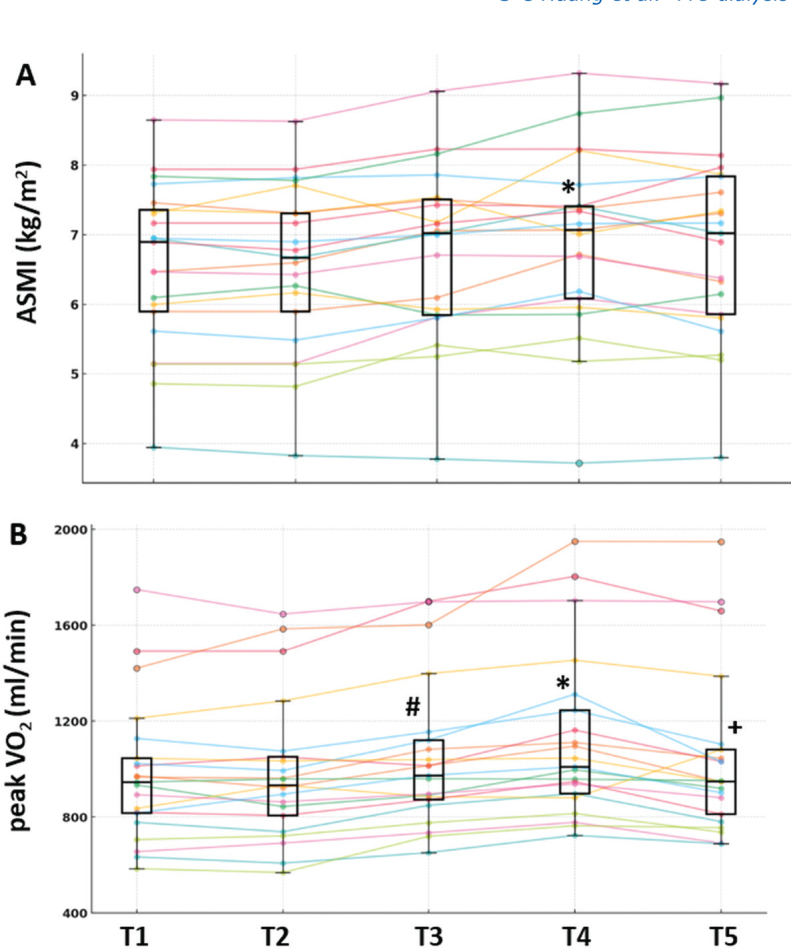
**Line graphs depicting individual values of (A) ASMI, and (B) peak V̇ O_2_**. Box plots illustrate the distribution of these variables across T1 to T5. *T2 vs T4, #T2 vs T3, +T4 vs T5, *p*-value < 0.05, pairwise comparison in mixed model of repeated measurements. ASMI: appendicular skeletal muscle mass index.

Normality tests indicated that the residuals of all key variables with significant findings in the mixed-model repeated measures analysis did not significantly deviate from normality, including 5 representative variables (ASMI, 60°PT, peak V̇O_2_, PCS, and MCS) (Appendix S1; S3). ASMI, lean mass of both legs, and trunk increased at T4 compared with T2, with no significant decline at T5 (Table II, Fig. 2A). The median IPT% and 60°PT predicted were 52% and 54% (20), indicating a substantial reduction in quadriceps strength in the maintenance HD population. Following training, quadriceps strength exhibited a pattern of change similar to that of muscle mass. While IPT increased after 36 training sessions, significant improvements in 60°PT, 120°PT, and the last third of 120°work were observed only after 72 training sessions. These improvements declined at T5, returning to near T2 levels (Table II).

**Table II:** DXA, IsoK strength, HGS, and CPET

Parameters	T1 Median (IQR)	T2 Median (IQR)	T3 Median (IQR)	T4 Median (IQR)	T5 Median (IQR)	*p*-value, T1 vs T2	*p*-value, T2 vs T3	*p*-value, T2 v. T4	*p*-value, T3 vs T4	*p*-value, T4 vs T5	*#p*-value, overall, fixed effect
DXA											
ASMI	6.69 (5.83,7.39)	6.64 (5.80,7.41)	7.01 (5.84,7.52)	7.04 (6.06,7.49)	6.96 (5.85,7.85)	1.000	0.060	< 0.001*	0.387	1.000	< 0.001*
Lean_total, 100 g	444 (337,5054)	436 (328,521)	434 (3358510)	443 (358,523)	431 (333,510)	1.000	1.000	0.300	0.431	0.446	0.112
Lean_Rt arm, 100 g	25 (18,29)	25 (19,30)	26 (18,32)	26 (18,32)	26 (19,32)	1.000	0.930	0.996	1.000	1.000	**0.021**
Lean_Lt Arm, 100 g	25 (16,31)	25 (17,30)	26 (17,29)	26 (18,31)	25 (17,30)	1.000	1.000	0.474	1.000	0.407	0.090
Lean_Rt Leg, 100 g	66 (50,75)	66 (50,77)	68 (53,76)	69 (58,81)	70 (53,79)	1.000	0.370	< 0.001*	< 0.001*	0.522	< 0.001*
Lean_Lt Leg, 100 g	63 (52,79)	63 (52,80)	64 (52,80)	65 (53,81)	63 (53,81)	1.000	1.000	0.011*	0.551	1.000	< 0.001*
Lean_trunk, 100 g	209 (166,251)	212 (169,260)	212 (171,258)	217 (175,264)	220 (167,25	1.000	1.000	0.010*	0.103	1.000	0.003*
Total body fat, %	33.0 (30.1,36.0)	33.1 (29.9,37.5)	33.6 (30.3,36.7)	32.6 (30.5,37.2)	32.8 (29.5,35.9)	1.000	1.000	1.000	1.000	1.000	0.656
Isokinetic quadriceps strength											
HGS, kg	28. (18.5,37.9)	26.9 (19.7,38.1)	27.9 (20.2,37.8)	29.4 (20.8,37.8)	29.6 (21.5,38.5)	1.000	1.000	1.000	1.000	1.000	0.613
IPT, N-M	78 (67,99)	79 (67,101)	92 (76,113)	93 (81,11)	84 (74,101)	1.000	**0.001**	< 0.001*	0.538	< 0.001*	< 0.001*
60°PT, N-M	71 (55,91)	70 (61,95)	76 (66,96)	87 (70,106)	70 (59,98)	0.393	0.440	< 0.001*	0.110	< 0.001*	< 0.001*
120°PT, N-M	57 (47,83)	64 (50,85)	68 (50,87)	77 (59,89)	66 (51,86)	0.383	0.274	< 0.001*	0.040*	0.04*	< 0.001*
120°total work, J	797 (572,1073)	847 (530,1049)	898 (585,1230)	978 (582,1105)	921 (623,1029)	1.000	0.830	0.770	1.000	1.000	0.236
120°work first third, J	301 (241,455)	347 (211,400)	351 (218,484)	350 (217,404)	340 (213,429)	1.000	1.000	1.000	1.000	1.000	0.773
120°work last third, J	201 (141,270)	229 (130,271)	230 (170,300)	247 (156,316)	232 (165,265)	1.000	0.067	0.037*	1.000	0.313	0.002*
120°work fatigue, %	37 (24,45)	38 (29,40)	32 (14,41)	30 (17,38)	37 (30,43)	1.000	0.471	1.000	1.000	1.000	0.150
CPET,											
Peak WR, watt	69 (54,82)	69 (55,82)	72 (60,85)	79 (64,104)	69 (55.76)	1.000	**0.004**	< 0.001*	< 0.001*	< 0.001*	< 0.001*
Peak VO_2_, ml/min	939 (806,1065)	927 (788,1056)	966 (858,1129)	1003 (893,1261)	947 (804,1086)	1.000	**0.025**	< 0.001*	**0.003**	**0.002**	< 0.001*
Peak VO_2_, ml/min/kg	14.8 (12.7,16.6)	15.1 (12.3,16.1)	15.6 (13.0,17.5)	17.1 (14.5,18.9)	15.1 (12.7,17.4)	1.000	**0.033**	< 0.001*	< 0.001*	< 0.001*	< 0.001*
VAT, ml/min	563 (484,639)	556 (473,633)	580 (515,677)	618 (547,764)	576 (502,651)	1.000	**0.025**	< 0.001*	< 0.001*	**0.002**	< 0.001*
VAT/BW, ml/min/kg	8.9 (7.6,9.9)	9.1 (7.4,9.7)	9.4 (7.8,10.5)	10.3 (8.6,11.4)	9.4 (7.8,10.5)	1.000	**0.049**	< 0.001*	< 0.001*	**0.001**	< 0.001*
Resting HR/min	77 (72,90)	78 (70,90)	76 (67,84)	75 (68,80)	76 (68,83)	1.000	0.817	0.466	1.000	1.000	0.028*
Peak HR/min	114 (102,131)	115 (98,126)	109 (101,132)	119 (102,131)	115 (96,130)	0.212	1.000	1.000	1.000	0.841	0.067
Resting SBP, mmHg	142 (128,153)	130 (115,151)	137 (114,146)	127 (115,155)	133 (114,151)	1.000	1.000	1.000	1.000	1.000	0.552
Peak SBP, mmHg	174 (157,183)	171 (147,182)	179 (155,194)	176 (161,196)	162 (147,191)	1.000	1.000	1.000	1.000	0.635	0.281

IQR: interquartile range; ASMI: appendicular skeletal muscle mass index; BF: breathing frequency; BW: bodyweight; CPET: cardiopulmonary exercise testing; DXA: dual-energy X-ray absorptiometry; EqCO_2_ nadir: the smallest value of ventilatory equivalent for CO_2_; HGS: hand grip strength; HR: heart rate; IPT: isometric peak torque; IsoK: isokinetic dynamometry; N-M: Newton-meter; pred: predicted; PT: peak torque; SBP: systolic blood pressure; VAT: ventilatory anaerobic threshold; V̇ O_2_: oxygen consumption; V̇ CO_2_: CO_2_ production; V̇ _E_: ventilation; WR: work rate. *P*-values less than 0.05 are highlighted in bold.

In this cohort, predicted peak V̇O2 was 60 ± 16%. Peak V̇O2 and VAT significantly increased from T2 to T4 (*p* < 0.001), with mean peak V̇O2 improvements of 0.8 ± 1.0 and 2.2 ± 1.5 ml/min/kg after 36 and 72 sessions, respectively. As the minimal clinically important difference is 1.5 mL/min/kg in patients with chronic kidney disease (21), only 72 sessions achieved meaningful gains in CRF. From T4 to T5, significant declines were observed in peak WR, peak V̇O2, V̇O2/BW, VAT, and VAT/BW (all p < 0.001) (Table II, Fig. 2B).

The IPAQ results showed that strenuous activity, moderate activity, walking, and TPA increased significantly during the training phase (T3, T4) but declined at T5, almost returning to pre-training levels (T1, T2) (Table III).

**Table III:** KDQOL (SF-36 and ESRD-related), MNA, and IPAQ

Parameters	T1 Median (IQR)	T2 Median (IQR)	T3 Median (IQR)	T4 Median (IQR)	T5 Median (IQR)	*p*-value, T1 vs. T2	*p*-value, T2 vs T3	*p*-value, T2 vs T4	*p*-value, T3 vs T4	*p*-value, T4 vs T5	*#p*-value, overall, fixed effect
KDQOL (SF-36)											
Physical functioning	65.0 (47.5,75.0)	60.0 (37.5,75.0)	70.0 (50.0,80.0)	70.0 (63.8,82.5)	67.5 (43.8,75.0)	1.000	0.806	0.735	1.000	1.000	**0.374**
Role physical	0.0 (0.0,50.0)	0.0 (0.0,31.3)	100.0 (43.8,100.0)	50.0 (0.0,75.0)	50.0 (0.0,75.0)	1.000	< 0.001*	0.001*	**0.026**	1.000	< 0.001*
Pain	67.5 (52.5,77.5)	70.0 (56.9,77.5)	67.5 (45.0,100.0)	77.5 (67.5,90.0)	77.5 (45.0,80.0)	1.000	1.000	0.001*	0.108	0.012*	< 0.001*
General health	40.0 (35.0,55.0)	47.5 (35.0,56.3)	55.0 (40.0,70.0)	45.0 (40.0,60.0)	50.0 (40.0,65.0)	1.000	0.181	0.767	1.000	1.000	0.027*
Emotional well-being	64.0 (55.0,72.0)	62.0 (48.0,72.0)	64.0 (56.0,80.0)	76.0 (68.0,80.0)	64.0 (63.0,76.0)	1.000	0.169	< 0.001*	0.233	0.169	< 0.001*
Role emotional	0.0 (0.0,100.0)	0.0 (0.0,33.3)	100.0 (66.7,100.0)	100.0 (66.7,100.0)	83.3 (58.3,100.0)	0.976	< 0.001*	< 0.001*	1.000	1.000	< 0.001*
Social function	75.0 (62.5,78.1)	75.0 (46.9,78.1)	75.0 (62.5,90.6)	87.5 (75.0,100.0)	75.0 (62.5,100.0)	1.000	1.000	0.057	1.000	1.000	**0.070**
Energy fatigue	47.5 (40.0,57.5)	45.0 (33.8,56.3)	50.0 (50.0,60.0)	57.5 (50.0,60.0)	50.0 (45.0,60.0)	0.606	0.068	0.010*	1.000	0.849	0.014*
PCS	35.1 (31.3,39.8)	35.6 (29.0,40.1)	42.6 (37.4,47.4)	40.3 (31.5,48.1)	40.6 (32.5,42.6)	1.000	< 0.001*	0.162	0.254	1.000	< 0.001*
MCS	46.9 (41.1,52.8)	45.6 (39.4,50.3)	50.9 (46.1,55.3)	57.2 (51.2,61.4)	48.5 (45.0,57.9)	1.000	**0.012**	< 0.001*	0.003*	< 0.001*	< 0.001*
KDQOL (ESRD-related)											
Symptom problem list	75 (68,86)	75 (72,82)	77 (75,88)	81 (79,93)	83 (75,84)	1.000	0.026*	< 0.001*	0.875	1.000	< 0.001*
Effects of kidney disease	56 (48,63)	56 (36,60)	69 (65,70)	78 (71,88)	66 (56,81)	1.000	0.003*	< 0.001*	0.064	0.001*	< 0.001*
Burden of kidney disease	22 (13,38)	25 (19,39)	50 (44,52)	50 (48,69)	50 (36,50)	1.000	< 0.001*	< 0.001*	1.000	0.028*	< 0.001*
Work status	50 (38,50)	50 (50,50)	50 (50,50)	50 (50,50)	50 (0,50)	1.000	1.000	1.000	1.000	0.275	0.023*
Cognitive function	83 (72,88)	77 (73,88)	87 80,100)	87 (80,100)	90 (80,93)	1.000	0.037*	0.001*	1.000	1.000	0.001*
Quality of social interaction	60 (53,80)	73 (60,87)	80 (73,83)	87 (73,87)	7 (67,95)	1.000	0.250	0.101*	1.000	1.000	0.001*
Sleep	59 (50,66)	55 (50,68)	64 (58,71)	60 (53,71)	68 (59,75)	1.000	0.07	0.850	1.000	0.711	0.002*
Social support	67 (50,83)	50 (50,71)	67 (67,100)	67 (67,100)	75 (67,100)	1.000	0.016*	0.001*	1.000	1.000	0.001*
Dialysis staff encouragement	75 (50,75)	75 (50,78)	75 (75,100)	100 (94,100)	75 (72,100)	1.000	0.053	<0.001*	0.033*	0.086	< 0.001*
Overall health	50 (40,60)	50 (50,53)	55 (50,60)	50 (40,70)	50 (40,60)	1.000	1.000	1.000	1.000	1.000	0.827
Patient satisfaction	83 (67,100)	67 (67,83)	83 (67,100)	83 (83,100)	83 (83,100)	0.109	0.057	0.001*	1.000	1.000	< 0.001*
MNA											
Screen score	14 (13,14)	14 (13,14)	14 (13,14)	14 (13,14)	14 (12,14)	1.000	1.000	0.741	1.000	1.000	0.297
Assessment score	12 (10,13)	12 (10,14)	13 (11,14)	13 (12,13)	13 (11,13)	1.000	0.373	0.505	1.000	1.000	0.025*
Total score	25 (23,27)	26 (22,28)	26 (25,28)	26 (25,27)	26 (24,27)	1.000	1.000	0.217	1.000	1.000	0.26
IPAQ											
Strenuous, min/week	0 (0,12.5)	0 (0,20)	30 (0,70)	30 (0,93)	0 (0,22.5)	1.000	**< 0.001**	**< 0.001**	1.000	**< 0.001**	**< 0.001**
Moderate, min/week	15 (0,65)	28 (0,83)	143 (90,255)	165 (90,278)	25 (0,90)	1.000	**< 0.001**	**< 0.001**	1.000	**< 0.001**	**< 0.001**
Walk, min/week	80 (53,135)	100 (53,135)	205 (115,375)	240 (115,420)	150 (60,360)	1.000	**< 0.001**	**< 0.001**	1.000	**0.047**	**< 0.001**
Sit, min/week	2180 (1680,3570)	2310 (1628,3570)	2520 (1485,3360)	2310 (1483,3360)	2520 (1680,3360)	1.000	1.000	1.000	1.000	1.000	0.556
TPA, min-METs/week	448 (303,812)	528 (330,812)	1741 (1097,2646)	2185 (1097,2925)	773 (323,1632)	1.000	**< 0.001**	**< 0.001**	1.000	**< 0.001**	**< 0.001**

IQR: interquartile range; ESRD: end-stage renal disease; IPAQ: International Physical Activity Questionnaire; KDQOL: Kidney Disease Quality of Life; MCS: mental component score of SF-36; MNA: mini-nutritional assessment; PCS: physical component score of SF-36; SF-36: 36-Item Short Form Health Survey; TPA: total physical activity. *P*-values less than 0.05 are highlighted in bold.

KDQOL improved in the domains of role physical, emotional well-being, role emotional, and energy/fatigue, with gains in both PCS and MCS scores. Regarding end-stage renal disease-targeted areas, KDQOL showed improvements in the domains of symptom/problem list, effects of kidney disease, burden of kidney disease, quality of social interaction, and dialysis staff encouragement. These benefits persisted during the 3-month follow-up (see Table III).

Mini Nutritional Assessment (MNA) scores remained consistent, with only minor increases observed across stages (*p*-value=0.025, fixed effect, for assessment scores) (see Table III).

No significant differences were observed in plasma levels of inflammatory or non-inflammatory cytokines (IL-1β, IL-6, IL-10, IL-12p70, and TNFα) between the pre- and post-training or detraining phases (Table IV). White blood cell differentials and inflammatory ratios (NLR, PLR) did not exhibit significant differences between stages (Appendix S1; S2).

**Table IV:** Inflammatory cytokines

Parameter	T1 Median (IQR)	T2 Median (IQR)	T3 Median (IQR)	T4 Median (IQR)	T5 Median (IQR)	*p*-value, T1 vs T2	*p*-value, T2 vs T3	*p*-value, T2 vs T4	*p*-value, T3 vs T4	*p*-value, T4 vs T5	*p*-value, overall, fixed effect
IL-12p, pg/mL	0.54 (0.08,0.90)	0.70 (0.15,1.05)	0.41 (0.15,0.85)	0.22 (0.08,0.95)	0.81 (0.15,1.25)	1.000	1.000	1.000	1.000	1.000	0.827
TNF, pg/mL	0.18 (0.14,0.67)	0.17 (0.15,0.21)	0.18 (0.15,0.51)	0.15 (0.11,0.29)	0.17 (0.14,0.22)	0.985	0.698	1.000	1.000	1.000	0.194
IL-10, pg/mL	0.82 (0.10,2.05)	0.71 (0.10,1.41)	0.85 (0.44,1.44)	0.82 (0.46,1.50)	1.06 (0.17,1.85)	1.000	1.000	1.000	1.000	1.000	0.472
IL-6, pg/mL	3.07 (1.06,5.95)	3.36 (2.23,6.18)	2.84 (2.01,6.08)	2.82 (0.51,7.22)	2.85 (1.21,6.20)	1.000	1.000	1.000	1.000	1.000	0.393
IL-1β, pg/mL	1.82 (1.04,2.37)	2.18 (1.41,2.53)	1.47 (1.01,2.22)	1.50 (0.98,2.22)	1.66 (0.57,2.23)	1.000	1.000	1.000	1.000	1.000	0.877
IL-8, pg/mL	14.10 (9.44,18.29)	14.48 (9.64,19.00)	15.25 (7.48,21.22)	13.26 (8.82,18.82)	14.09 (9.35,17.14)	1.000	1.000	1.000	1.000	1.000	0.604

Mixed models for repeated measures and Bonferroni pairwise comparison.

IQR: interquartile range.

## DISCUSSION

To our knowledge, this is the first study to implement a conventional structured aerobic and resistance training programme for patients undergoing maintenance haemodialysis delivered prior to their dialysis sessions. The training phase lasted 6 months. It comprehensively assessed the effects of PDE on multiple dimensions of physical fitness – using DXA, isokinetic testing, and CPET – and also evaluated QOL and inflammatory markers. The study also tracked changes 3 months after the cessation of training. The main findings were summarized as follows. (I) Collectively, CRF, isokinetic quadriceps strength, and muscle mass improved following training but the progression was slow. Clinically or statistically significant improvements were generally observed after 72 training sessions. In contrast, 36 sessions did not lead to statistically significant changes in muscle mass or quadriceps strength. Meanwhile, considering that the minimal clinically important difference for peak V̇O_2_ in patients with chronic kidney disease is 1.5 mL/kg per minute (21), 36 training sessions were insufficient to achieve this threshold. Instead, 72 training sessions were required to elicit clinically meaningful adaptations in CRF. (II) The compliance rate for the 6-month training period was as high as 92%. (III) Following in-hospital training, median TPA was substantially reduced from 2,185 to 773 minimum Metabolic Equivalents (min-METs)/week. The lack of physical activity is associated with a rapid decline in CRF and quadriceps strength to approximately pre-training levels within just 3 months. Nonetheless, muscle mass appeared to be relatively preserved after detraining. (IV) Generic health-related QOL was also improved in both PCS and MCS. Chronic kidney disease-related QOL also improved in several domains following training including symptom problem list, effects of kidney disease, burden of kidney disease, quality of social interaction, and dialysis staff encouragement. (V) Plasma inflammatory cytokine levels were not different between pre- and post-training or detraining, suggesting that PDE has limited efficacy in altering systemic inflammatory responses. (VI) TPA increased significantly during the training phase but declined to pre-training levels during the follow-up phase.

Previous meta-analyses have confirmed the positive effects of supervised exercise training on muscle strength, muscle mass, and aerobic capacity. However, most of the included studies involved exercise performed during haemodialysis (7, 22, 23). Based on the present findings, compared with intradialytic training, PDE offers advantages for higher-intensity aerobic and whole-body resistance training, and appears to be associated with good adherence and robust improvement in MCS. The following section specifically summarizes the limited evidence on exercise interventions conducted during the pre-dialysis period.

### Pre-dialytic training

Pre-dialysis volume overload is defined as the sum of interdialytic weight gain and residual post-dialysis volume overload (24). While there is no published evidence supporting an absolute contraindication to exercise based on an interdialytic weight gain exceeding a certain threshold, concerns remain that excessive interdialytic weight gain may affect resting blood pressure and heart rate levels, and potentially negatively affect haemodynamics during exercise (25). Our previous study demonstrated that pre-dialysis setting generally did not impact performance during CPET, isokinetic muscle strength, or endurance compared with non-HD days. Additionally, changes in blood pressure during HD were not affected by exercise conducted 1–2 h before HD (26).

To the best of our knowledge, only 5 studies have investigated hospital-based exercise training before HD. First, a randomized study showed that 24-week pre-dialysis resistance training (RT) (1 h before HD, 3×/week) reduced sarcopenia and improved strength, body composition, functional performance, and biomarkers (TNFα, IL-6, anaemia). Dropout for non-medical reasons was 12.5% (9 out of 72) (13). In the second study, HD patients were randomized to exercise or control. The 12-week programme combined pre-dialysis RT (1.86×/week, ~17 min) and intradialytic cycling (2.73×/week, ~50 min). It improved lower-limb strength, but not aerobic capacity. Dropout for non-medical reasons was 8.6% (5 out of 60 participants) (10). The third study showed that combining oral nutrition and RT (~70% 1–repetition maximum, 30 min before HD, 3×/week for 6 months) improved muscle strength, with a 13% dropout rate (11). The fourth study found that 12 weeks of moderate-intensity RT (3×/week before HD) enhanced body composition, fitness, and QOL, with a 9% dropout rate (12). The fifth study reported that an 8-week virtual reality programme (40 min/session, 3×/week during pre-dialysis waiting) improved physical fitness in HD patients. Of 24 participants, only 1 (4.1%) dropped out due to emergency surgery (14).

Collectively, no serious exercise-related adverse events were reported in the 5 cited studies or the present study, across ~12,275 person-sessions. Notably, the dropout rates in the training group for non-medical reasons were low (0–13%). These findings suggest that pre-dialysis exercise is safe, improves physical fitness, QOL, and has high compliance. Pre-dialysis exercise can thus be an alternative model for patients undergoing maintenance HD. Exercise training models should aim to provide patients with greater autonomy in selecting their preferred training approach. A flexible exercise prescription is beneficial for adherence (26).

The present study demonstrated no differences in plasma levels of either inflammatory or non-inflammatory cytokines between the pre- and post-training or detraining periods. In line with our findings, a network meta-analysis reported that aerobic exercise, resistance exercise, and stretching exercise neither increased nor decreased IL-6. Similar findings were obtained for C-reactive protein (27). These findings indicate that exercise does not elevate mortality risk via inflammatory responses, yet it offers no clear benefit in mitigating inflammation or reducing inflammation-related cardiovascular mortality.

### Quality of life

Previous studies indicate that haemodialysis patients have a reduced ability to perform activities of daily living compared with age-matched controls. Even after adjusting for comorbidities, ESRD patients still experience a lower QOL (2). The present study demonstrated that both the physical and mental composites of the 36-Item Short Form Health Survey (SF-36) improved after exercise training, which aligns with the previous literature that intradialytic aerobic and resistance exercises are effective in improving both PCS and MCS and depression symptoms in maintenance haemodialysis patients (22, 29). Given that the minimal clinically important difference for the SF-36 scales is between 3 and 5 points (30, 31), we consider the effect of PDE on PCS (median T2 vs T4: 36 vs 40) to be small; the effect on MCS is apparent (median T2 vs T4: 46 vs 57). We speculate that the more pronounced improvement in MCS might be associated with group-based training and encouragement from the rehabilitation team. The effect is significant in the domains of role physical, emotional well-being, and role emotional. Role physical assesses the extent to which physical health problems limit daily work or other everyday activities. The improvement could relate to the improvement of aerobic capacity and quadriceps strength. Role emotional assesses the extent to which emotional problems limit daily work or other everyday activities. Emotional well-being assesses an individual’s emotional state, including aspects such as anxiety, depression, calmness, and happiness (32).

Regarding the kidney-disease-specific QOL, improvement was noted primarily in the following domains: symptom/problem list, effects of kidney disease, quality of social interaction, burden of kidney disease, and dialysis staff encouragement. Symptom/problem list refers to a section that evaluates the frequency and severity of common symptoms and problems associated with chronic kidney disease and dialysis. This domain assesses a range of physical and emotional symptoms. For example, significant improvement was observed in question 15a: drinking water restriction. Due to excessive sweating during exercise training, water restriction may be eased to some extent. Effects of kidney disease assesses the impact of kidney disease on a patient’s daily life, including mobility, work, travel, sleep, and appetite. Quality of social interaction evaluates the quality of a patient’s social activities, including interactions with family and friends, as well as the frequency and satisfaction of social engagement. Open-ended qualitative data revealed that some participants tended to withdraw from social relationships before joining the programme. After participation, however, they reported engaging in conversations with other patients during exercise training and developing an enjoyable social network. Burden of kidney disease measures the perceived burden of kidney disease on a patient, including its impact on time, emotions, and personal life. After participating in the programme, they felt that kidney disease placed less of a burden on their daily functioning and emotional well-being.

### Detraining

This study showed that CRF and quadriceps strength was not sustained during the follow-up phase, while muscle mass seemed relatively resistant to detraining. This order of change in physical fitness is generally consistent with previous studies. It was reported that physical deconditioning for 3–8 weeks in highly trained individuals results in a 20% reduction in peak V̇O_2_ (33). Similarly, in 48 patients with metabolic syndrome, a 4-month aerobic interval training programme was followed by a ~10% decline in peak V̇O_2_ 1 month after cessation (34). Generally, training-induced gains are most often completely reversed when training is stopped for a period longer than 4 weeks. The decline in CRF is primarily attributed to a rapid reduction in blood volume, which can occur within as little as 2 days, followed by cardiovascular changes such as reduced left ventricular volume (35). As to the strength decline after detraining, 10 elderly women (67 ± 5 years) completed 12 weeks of strength training followed by 12 weeks of detraining, resulting in a 17.8% decline in knee extensor strength (36). Another 10 women (mean age: 82.8 years) were reassessed 1 year after completing a strength training programme, show-ing 24.4%–68.3% losses in dynamic and isometric strength (37). This decline is mainly attributed to the loss of neural adaptations, including decreased motor unit recruitment and impaired neuromuscular control, along with reduced muscle fibre cross-sectional area (36). On the other hand, muscle mass is relatively resistant to detraining, remaining generally stable for 12–24 weeks before experiencing a marked reduction beyond 31 weeks in older adults. This decline is primarily due to an imbalance between decreased protein synthesis and increased protein breakdown (38). Overall, these findings demonstrate the varying sensitivity of each component to the absence of training stimuli. The results also highlight the very limited carry-over effect of training in the HD population, underscoring the need for an effective home programme.

### Limitations

First, the present study did not include patients with heart failure or non-ambulatory patients. The physical condition of the study population was thus relatively better than that of the overall HD population, particularly considering that approximately 40% of HD patients are non-ambulatory (39). Therefore, the findings of this study cannot be generalized to all patients undergoing chronic HD. Second, the measurement of physical activity was based on a questionnaire rather than objective assessment tools, which may potentially affect validity. However, the accuracy of the IPAQ is considered acceptable (40). Third, the study did not compare exercise interventions delivered at different time points (during HD, on non-dialysis days, and through PDE), and future studies are warranted to address this issue. Fourth, the lack of a parallel control group may have introduced potential time-related confounding, and the possibility of a Hawthorne effect due to increased attention cannot be excluded. Fifth, outcome assessors were not blinded, which is particularly relevant for QOL measures. Finally, the sample size estimation in this study was not based on a formal *a priori* power calculation, which may affect the robustness and generalizability of the findings. Instead, the decision on sample size was guided by prior literature on PDE exercise training, in which the number of participants completing the intervention ranged from 10 to 23 (11–14), as well as our previous studies of exercise training in patients with coronary artery disease (41) and stroke (42). Although only 22 participants completed the present 1-year protocol, clinically meaningful improvements were observed, supporting the potential value of the intervention.

### Conclusion

Pre-dialysis exercise training is safe, and associated with high adherence among patients undergoing maintenance haemodialysis who are independently ambulatory and have no history of heart failure. A 6-month, 72-session combined aerobic and resistance programme performed 1–2 h before dialysis improves CRF, muscle strength, muscle mass, and both generic and kidney disease-specific QOL. However, gains in CRF and strength decline within 3 months post-training. Therefore, PDE may offer an alternative exercise model for HD patients; however, an effective maintenance programme is essential to sustain its benefits.

## Supplementary Material


