# Supplementary material for: FROM GAINS TO DECLINE: EFFECTS OF STRUCTURED PRE-DIALYTIC EXERCISE TRAINING AND DETRAINING ON PHYSICAL FITNESS, QUALITY OF LIFE, AND INFLAMMATION IN HAEMODIALYSIS PATIENTS
Source: J Rehabil Med. 2025 Oct 22;57:44067. doi: 10.2340/jrm.v57.44067 (PMC12666888; doi:10.2340/jrm.v57.44067)
Supplement: Supplementary file 1 [file JRM-57-44067-s1.pdf]

Supplementary material has been published as submitted. It has not been copyedited, or typeset by Journal of Rehabilitation Medicine

Appendix S1

S1. Methodology

2.3. Nutrition Program

After T2 assessment, the participants were first required to complete an estimated food record in the past 7 days. A dietitian then conducted a nutritional assessment to provide a comprehensive evaluation and to estimate the required amount and relative proportions of macronutrients. Based on this assessment, food-based dietary advice was given. Each participant received a logbook, and adherence to the dietary recommendations was monitored by the dietitian. For dialysis patients, the recommended energy and protein intake were 25–32 kcal/kg and 1.2–1.3 g/kg of body weight, respectively [1, 2]. After each training session, the participants were provided with a commercially available high-protein supplement (Red Cow Aiji®).

2.4. Cardiopulmonary exercise testing

A symptom-limited incremental exercise test was conducted on a calibrated bicycle ergometer (Ergoselect 150P, Germany) to assess aerobic fitness. The test began with an 1-minute warm-up at 10 watts, followed by a 10-watt-per-minute increase until exhaustion, targeting 60 ± 3 revolutions per minute (rpm). Breath-by-breath minute ventilation ($\dot{V}e$), oxygen consumption ($\dot{V}O_2$), and carbonic dioxide production ($\dot{V}CO_2$) were recorded using MasterScreen CPX (Cardinal-health Germany). Heart rate, arterial pressure, and oxygen saturation were monitored using a 12-lead electrocardiogram, an automatic blood pressure system (Tango, SunTech Medical, UK), and a pulse oximeter (Nonin Onyx 9500, USA), respectively. The test ended if the participants fell below 50 rpm, reached volitional fatigue, showed a peak $\dot{V}O_2$ plateau/decline despite continued exercise, or experienced cardiovascular events. Data were averaged every 15 seconds. Ventilatory anaerobic threshold (VAT) was determined using V-slope method and confirmed by non-linearity of $\dot{V}CO_2$ vs. $\dot{V}O_2$, increased $\dot{V}e$-$\dot{V}O_2$ ratio without a rise in $\dot{V}e$-$\dot{V}CO_2$ ratio, and rising end-tidal oxygen without declining end-tidal carbon dioxide [3].

2.5. Body composition

This study utilized a fan-beam DXA body composition analyzer (Lunar Prodigy; GE Healthcare, Madison, WI), with data analyzed by GE Encore 12.30 software. The analyzer was calibrated before use, and scans were conducted in standard mode. Results were automatically analyzed and verified by trained professionals. Radiologists ensured participants maintained consistent posture: lying centered on the machine with foam

bricks keeping their feet 15 centimeters apart and palms 3 centimeters from the torso. This process measured fat mass, muscle mass and appendicular skeletal muscle mass index (ASMI) [4].

2.6. Isokinetic dynamometry

Quadriceps peak torque (PT) was measured using a Biodex isokinetic dynamometer (System 4 Pro™; New York, NY, USA). Isometric PT (IPT) was measured at 45°, and concentric PT across a 90°–0° range. Participants exerted five maximal-effort repetitions at angular velocities of 0°/s, 60°/s, and 120°/s, with a 2-minute rest between sets. Tests with >10% variance were repeated [5]. After a 10-minute rest, participants performed a fatigue test at 120°/s, consisting of 20 maximal-effort concentric contractions. Tests with a variance greater than 15% were repeated [6]. Total work was calculated as the energy exerted during the 20 repetitions, and the fatigue index (%) measured the work decline in the final one third repetitions compared to the initial one third [7].

2.7. Hand grip strength

Hand grip strength (HGS) was measured using a dynamometer (Tsutsumi Company, Tokyo). Participants stood with palms facing their bodies, adjusting the grip for optimal force. They squeezed the dynamometer maximally for 3 seconds in two trials per hand, alternating sides. The highest value from each hand was averaged [8].

2.8. Hong Kong Chinese Kidney Disease Quality of Life

The KDQOL-SF™ v1.3 survey was used to assess quality of life in patients with chronic kidney disease in HK, tailored for Mandarin speakers with high responsiveness in this population [9]. It includes the 11-item Short Form Health Survey and 17 kidney disease-specific items. The former covers eight categories: physical functioning, role-physical, pain, general health, emotional well-being, role-emotional, social function, and energy/fatigue, summarized into physical component summary (PCS) and mental component summary (MCS) scores. The latter covers 11 domains: symptom/problem list, effects of kidney disease, burden of kidney disease, work status, cognitive function, quality of social interaction, sleep, social support, dialysis staff encouragement, overall health, and patient satisfaction, excluding the sexual function domain [10].

2.9 International Physical Activity Questionnaire (IPAQ)

The Taiwan version of the IPAQ [11] was used to evaluate participants' physical activity levels, including vigorous, moderate, walking activities, and sitting, over the past 7 days. Participants reported their engagement in vigorous activities (e.g., running), moderate activities (e.g., light cycling), and walking for commuting or leisure, along with sitting time for desk work or TV. Total physical activity (TPA) was calculated as: (vigorous activity time × 8) + (moderate activity time × 4) + (walking time × 3.3) [12].

2.10. Measurement of plasma inflammatory cytokines and white blood cell differential counts

Venous blood collection was performed by medical professionals at the hemodialysis center before hemodialysis (HD). A total of 3 mL of blood was drawn from the arteriovenous shunt of each participant and placed into tubes containing Sodium Heparin 158 USP. Plasma was separated by centrifugation at 1,300 × g for 15 min at 4°C within one hour of collection and then stored at -80°C until analysis. Interleukin (IL)-1 beta (β), IL-6, IL-8, IL-10, IL-12p70, and tumor necrosis factor-α (TNFα) concentrations were detected in plasma using the BD Cytometric Bead Array (CBA) Human Inflammatory Cytokines Kit (Becton-Dickinson) and analyzed with FCAP Array™ software [13].

2.11. Nutritional assessment

The MNA is a validated tool for assessing nutritional status, comprising two parts. The first part includes six questions for the screening score, while the second part includes twelve questions about dietary habits, self-perceived health, and nutritional status, forming the assessment score. These questions cover meal frequency, food variety, self-sufficiency in eating, and the impact of stress or illness. The total score is the sum of the screening and assessment scores [14].

References

1. Kopple JD. National kidney foundation K/DOQI clinical practice guidelines for nutrition in chronic renal failure. Am J Kidney Dis. 2001;37:S66-70.
2. Ikizler TA, Cuppari L. The 2020 Updated KDOQI Clinical Practice Guidelines for Nutrition in Chronic Kidney Disease. Blood Purif. 2021;50:667-671.
3. Whipp BJ, Ward SA, Wasserman K. Respiratory markers of the anaerobic threshold. Adv Cardiol. 1986;35:47-64.
4. Nana A, Slater GJ, Hopkins WG, Burke LM. Effects of exercise sessions on DXA measurements of body composition in active people. Med Sci Sports Exerc. 2013;45:178-185.
5. O'Connor RF, King E, Richter C, Webster KE, Falvey É C. No Relationship Between Strength and Power Scores and Anterior Cruciate Ligament Return to Sport After Injury Scale 9 Months After Anterior Cruciate Ligament Reconstruction. Am J Sports Med. 2020;48:78-84.
6. Hsiao CC, Chou CY, Fang JT, Chang SC, Liu KC, Huang SC. Cardiopulmonary Response to Acute Exercise before Hemodialysis: A Pilot Study. Kidney Blood Press Res. 2024;49:735-744.

7. McLeland KA, Ruas CV, Arevalo JA, Bagley JR, Ciccone AB, Brown LE, et al. Comparison of knee extension concentric fatigue between repetition ranges. Isokinetics and Exercise Science. 2016;24:33-38.
8. Huang SC, Yang LY, Chao YK, Chang WY, Tsao YT, Chou CY, et al. Improved functional oral intake and exercise training attenuate decline in aerobic capacity following chemoradiotherapy in patients with esophageal cancer. J Rehabil Med. 2024;18;56:jrm25906.
9. Chow SK, Tam BM. Is the kidney disease quality of life-36 (KDQOL-36) a valid instrument for Chinese dialysis patients? BMC Nephrol. 2014;15;15:199.
10. Hays RD, Kallich J, Mapes D, Coons S, Amin N, Carter W, et al. Kidney Disease Quality of Life Short Form (KDQOL-SF™), version 1.3: a manual for use and scoring. Santa Monica: RAND. 1997:7994.
11. Liou YM, Jwo CJ, Yao KG, Chiang LC, Huang LH. Selection of appropriate Chinese terms to represent intensity and types of physical activity terms for use in the Taiwan version of IPAQ. J Nurs Res. 2008;16:252-263.
12. Tomioka K, Iwamoto J, Saeki K, Okamoto N. Reliability and validity of the International Physical Activity Questionnaire (IPAQ) in elderly adults: the Fujiwara-kyo Study. J Epidemiol. 2011;21:459-465.
13. Lin SJ, Kuo ML, Hsiao HS, Lee PT. Azithromycin modulates immune response of human monocyte-derived dendritic cells and CD4(+) T cells. Int Immunopharmacol. 2016;40:318-326.
14. Cereda E. Mini nutritional assessment. Current Opinion in Clinical Nutrition & Metabolic Care. 2012;15:29-41.

S2. White blood cells differential count

parameters	unit	T1	T2	T3	T4	T5	*p*-value, T1 vs. T2	*p*-value, T2 vs. T3	*p*-value, T2 vs. T4	*p*-value, T3 vs. T4	*p*-value, T4 vs. T5	*p*-value, overall, fixed effect
**Neutrophil**	x10^3/µL	3.36(2.39,5.28)	3.3(2.64,4.21)	3.85(3.15,5.12)	3.44(2.69,4.43)	3.83(2.68,4.62)	1.000	1.000	1.000	1.000	1.000	0.570
**Lymphocyte**	x10^3/µL	1.33(1.02,1.61)	1.24(1.03,1.65)	1.27(1.06,1.54)	1.13(0.85,1.55)	1.22(0.79,1.56)	1.000	1.000	1.000	1.000	1.000	0.962
**monocyte**	x10^3/µL	0.31(0.25,0.4)	0.37(0.33,0.42)	0.42(0.36,0.45)	0.36(0.31,0.48)	0.32(0.31,0.44)	1.000	1.000	1.000	1.000	1.000	0.196
**NLR**		2.14(1.71,4.9)	2.62(2.19,3.4)	3.41(2.35,3.72)	3.3(2.11,4.48)	3.29(2.07,4.62)	1.000	1.000	1.000	1.000	1.000	0.672
**PLR**		144(113,253)	138(119,185)	146(113,209)	136(125,183)	145(124,163)	1.000	1.000	1.000	1.000	1.000	0.471

NLR: neutrophil lymphocyte ratio; PLR: platelet lymphocyte ratio

S3. Normality testing

Catalog

1. Normality Assessment of Residuals for All Key Variables With Significant Findings in the Mixed-Model Repeated Measures Analysis Using the Kolmogorov–Smirnov Test3

2. Normality Assessment of ASMI Residuals Using Q–Q Plot and Histogram With Normal Curve 5

3. Normality Assessment of 60° PT residuals Using Q–Q Plot and Histogram With Normal Curve 6

4. Normality Assessment of peak VO_2 (ml/min/kg) residuals Using Q–Q Plot and Histogram With Normal Curve 7

5. Normality Assessment of PCS residuals Using Q–Q Plot and Histogram With Normal Curve 8

6. Normality Assessment of MCS residuals Using Q–Q Plot and Histogram With Normal Curve 9

7. Conclusion. 10

Normality tests of residuals for all key variables with significant findings in the mixed model of repeated measures

Variable	unit	KS Statistic	**KS p-value**
ASMI	Kg/m^2	0.049	0.943
Lean_Rt arm	100g	0.090	0.313
Lean_Rt leg	100g	0.090	0.275
Lean_Lt leg	100g	0.120	0.079
Lean_trunk	100g	0.104	0.176
IPT	N-M	0.090	0.297
60°PT	N-M	0.083	0.406
120°PT	N-M	0.123	0.065
120°work last third	J	0.060	0.957
Peak WR	watt	0.089	0.329
Peak VO_2	ml/min	0.108	0.142
Peak VO_2/BW	ml/min/kg	0.060	0.807
VAT	ml/min	0.077	0.501
VAT/BW	ml/min/kg	0.062	0.767
Resting HR	/min	0.052	0.912
Role physical	-	0.068	0.670
Pain	-	0.068	0.670
General health		0.083	0.413
Emotional wellbeing	-	0.084	0.392
Role emotional	-	0.079	0.473
social function		0.083	0.413
Energy fatigue	-	0.071	0.615
PCS	-	0.074	0.558
MCS	-	0.065	0.710
Symptom problem list	-	0.100	0.207
Effects of kidney disease	-	0.090	0.311
Burden of kidney disease	-	0.088	0.339
Cognitive function	-	0.110	0.131
Quality of social interaction	-	0.084	0.406
sleep		0.083	0.413

Social support	-	0.084	0.397
Dialysis staff encouragement	-	0.098	0.225
Patient satisfaction	-	0.119	0.084
TPA	min-METs/week	0.095	0.255

KS: Kolmogorov-Smirnov Test

ASMI: appendicular skeletal muscle mass index; BW: body weight; IPT: isometric peak torque; N-M: Newton-meter; MCS: mental component score of SF-36; PCS: physical component score of SF-36; PT: peak torque; TPA: total physical activity; VAT: ventilatory anaerobic threshold; $\dot{V}O_2$: oxygen consumption; WR: work rate

2. residual of ASMI

■ QQ Plot

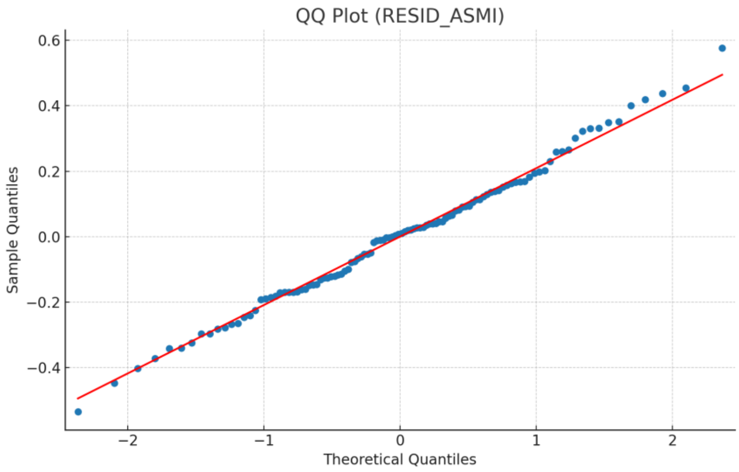

■ Histogram with Normal Curve

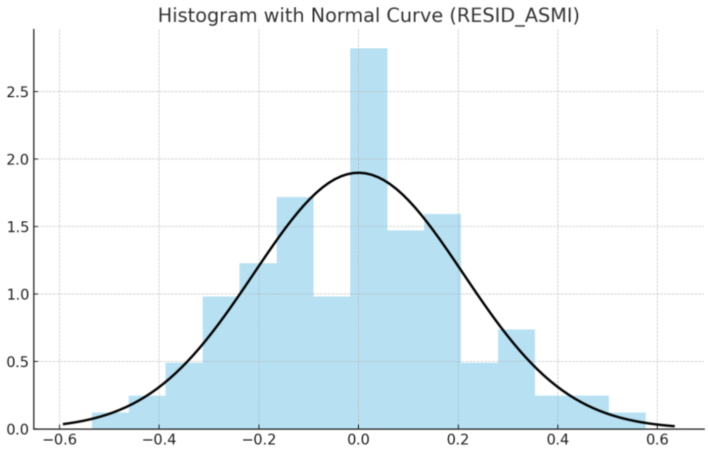

3. residual of 60° PT

■ QQ Plot

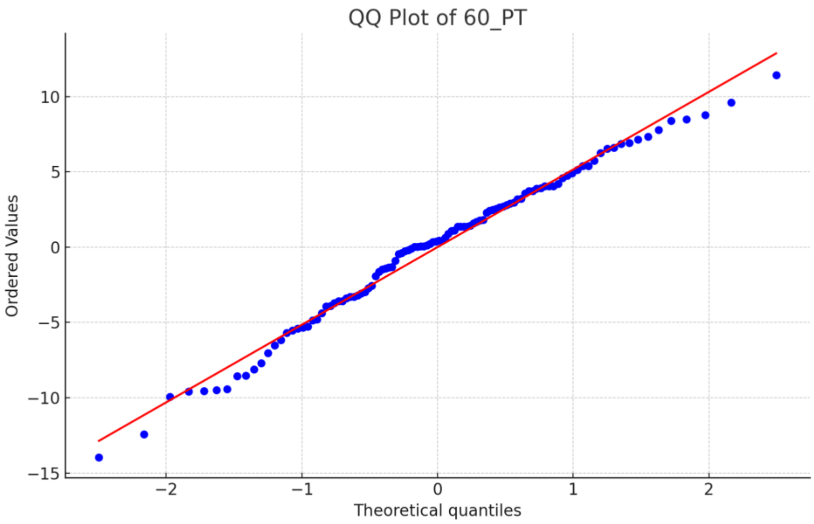

■ Histogram with Normal Curve

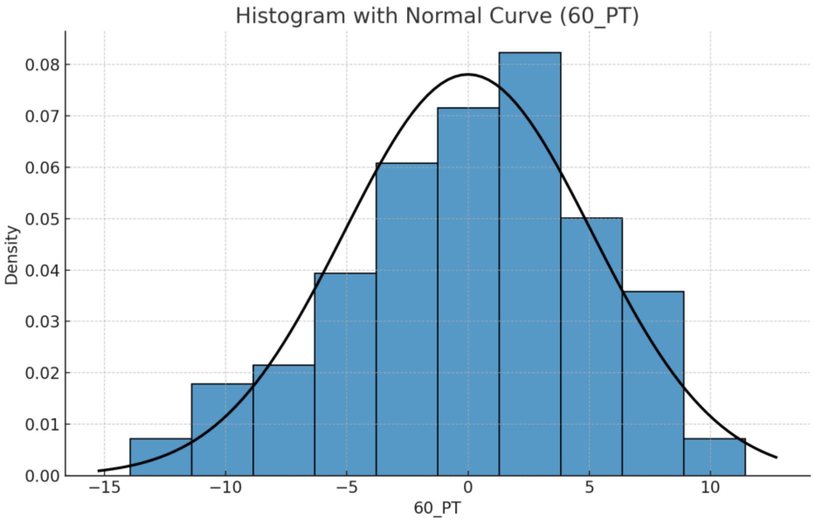

4. residual of peak VO_2 (ml/min/kg)

■ QQ Plot

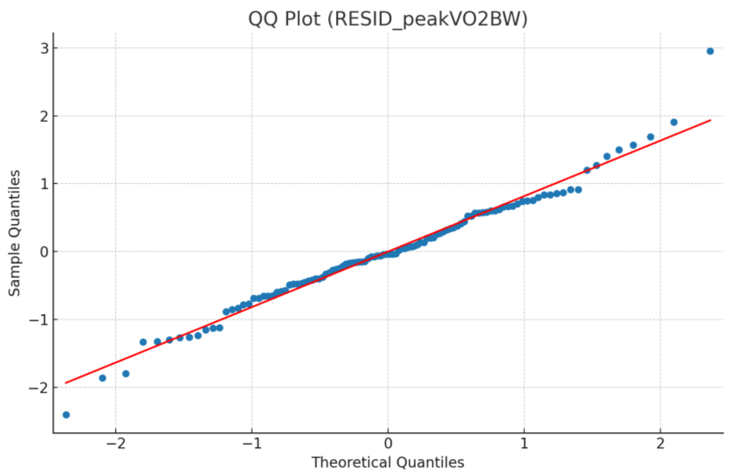

■ Histogram with Normal Curve

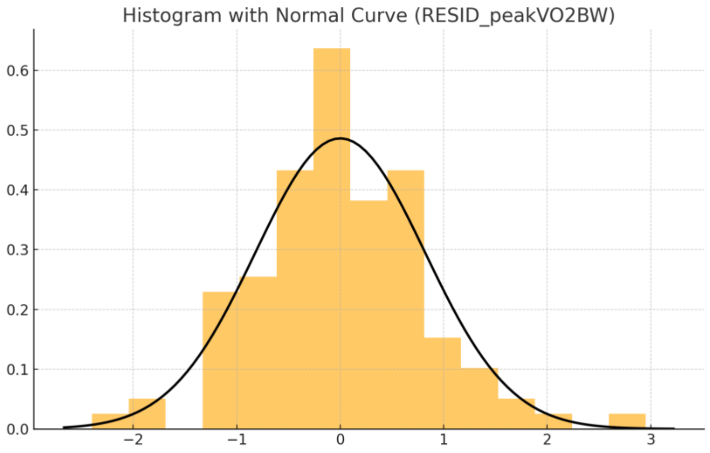

5. residual of PCS

■ **QQ Plot**

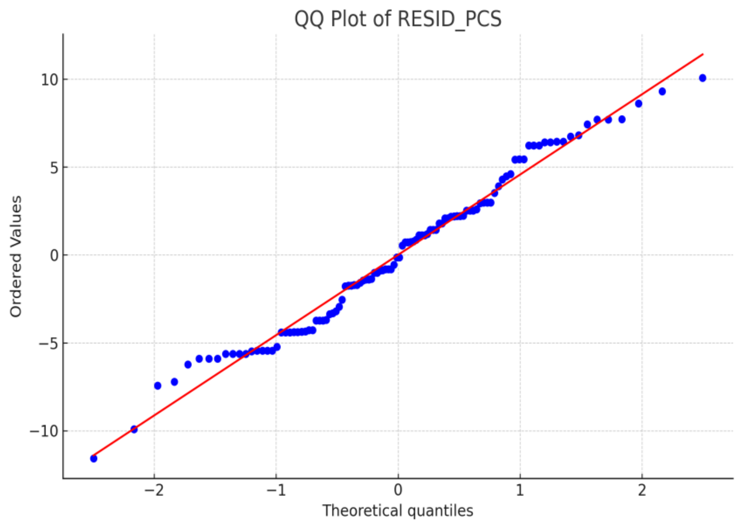

■ **Histogram with Normal Curve**

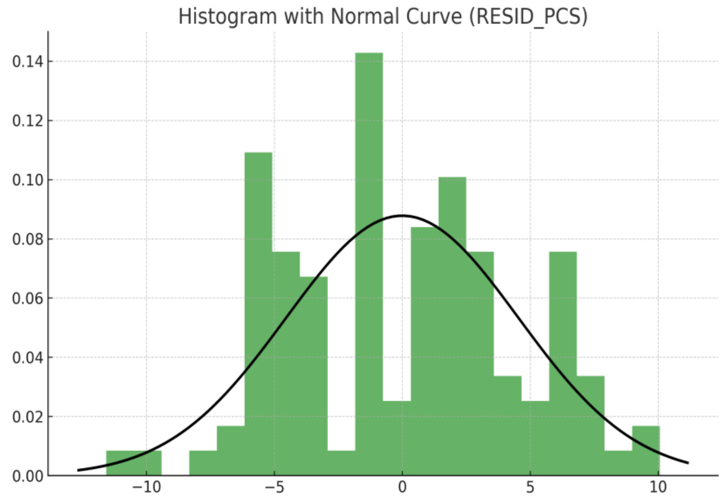

6. residual of MCS

■ **QQ Plot**

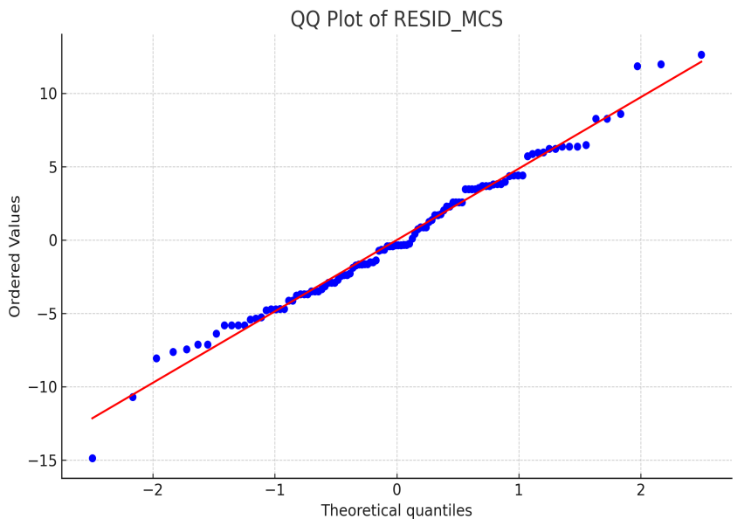

■ **Histogram with Normal Curve**

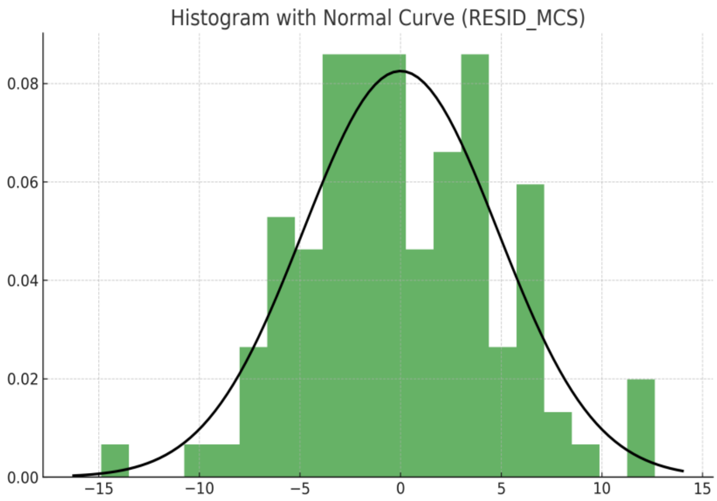

7. Conclusion:

Based on the above analyses (1–6), normality of residuals for all key variables with significant findings in the mixed-model repeated measures analysis (Tables 2 and 3) was assessed using the Kolmogorov–Smirnov test, which showed no significant deviations from normality. Furthermore, five representative variables (ASMI, 60°PT, peak $\dot{V}O_2$, PCS, and MCS) were additionally examined using Q–Q plots and histograms with normal curves, which also indicated no significant deviations from normality.
